# A child with factor V deficiency with a novel F5 gene mutation misdiagnosed as a left iliac fossa abscess: A case report

**DOI:** 10.1097/MD.0000000000040436

**Published:** 2024-11-15

**Authors:** Yifan Zhang, Lu Liu, Qin Guo, Yiyuan Li, Shuanghong Luo, Chaomin Wan, Yu Zhu

**Affiliations:** a Department of Pediatrics, West China Second Hospital, Sichuan University, Chengdu, People’s Republic of China; b Key Laboratory of Birth Defects and Related Diseases of Women and Children (Sichuan University), Ministry of Education, Chengdu, China; c NHC Key Laboratory of Chronobiology (Sichuan University), Chengdu, China.

**Keywords:** blood coagulation disorder, case report, clotting factors, congenital disorders, hematology

## Abstract

**Rationale:**

Congenital factor V deficiency is classified as a rare bleeding disorder that is expressed in an autosomal recessive manner and generally occurs in 1 in a million people. This disorder is accompanied by a variety of clinical manifestations, which can easily lead to misdiagnosis. This is the first report to identify the factor V gene variant c.2439delC (p.I814Lfs*23) in exon 13.

**Patient concerns:**

A 13-year-old boy was admitted with a suspected left iliac fossa abscess. He had been previously diagnosed with and underwent management for a left iliac fossa abscess at a local hospital. The patient was treated with antibiotics and underwent surgical excision; however, his left iliac fossa mass reappeared. Platelet count and function, prothrombin time, and activated partial thromboplastin time were all normal.

**Diagnoses:**

The patient was considered to have congenital factor V deficiency following the measurement of coagulation factor activity, and the diagnosis was confirmed by genetic testing.

**Interventions:**

The mass was diagnosed as an abscess and the patient was treated with antibiotics at the local hospital. Surgical resection was performed, after which the mass was identified as a hematoma. The patient was then transferred to our hospital for treatment with fresh frozen plasma (FFP) infusion.

**Outcomes:**

The left iliac fossa mass stopped growing and the coagulation function exhibited a significant improvement. At discharge, the patient was recommended to seek medical help before any surgical intervention or following trauma, and when a deep hemorrhage is identified, the patient should undergo timely infusion with FFP.

**Lessons:**

This case report presents a rare occurrence of congenital factor V deficiency resulting in a left iliac fossa hematoma mistaken for an abscess, which resulted in unnecessary antibiotic therapy and surgery. This case emphasizes that coagulation factor deficiency should be highly suspected for joint mass combined with coagulation dysfunction.

## 1. Introduction

Factor V deficiency is a rare bleeding disorder.^[1]^ Congenital factor V deficiency is expressed in an autosomal recessive manner. This disorder is characterized by various bleeding features; however, life-threatening bleeding has rarely been reported.^[2]^ The first step in diagnosing factor V deficiency is routine coagulation testing. In addition, genomic analysis can be used to confirm the diagnosis.^[3,4]^ Herein, we describe the case of a child with congenital factor V deficiency due to coagulation dysfunction combined with a joint mass which was mistaken for an abscess, resulting in unnecessary antibiotic and surgical therapy. The factor V gene variant c.2439delC (p.I814Lfs*23) in exon 13 was reported for the first time in this case.

## 2. Case report

The patient was a 13-year-old boy living in Sichuan Province, China. He was treated at a local hospital for a left iliac fossa mass combined with a left lower extremity movement disability. The mass was diagnosed as an abscess, and the patient was treated with antibiotics; subsequently, surgical resection was performed at a local hospital, and the mass was identified as a hematoma. On postoperative day 1, the mass reappeared in the left iliac fossa. Coagulation function was abnormal and manifested as a prolonged prothrombin time (PT) and activated partial thromboplastin time (APTT) and increased international normalized ratio (Table 1). The patient reported no history of treatment with specific drugs or exposure to poison or being bit by insects, snakes, and animals. He had no evidence of gingival bleeding, central nervous system bleeding, gastrointestinal bleeding, or hemorrhage. The patient had a history of childhood recurrent epistaxis. He was admitted to our hospital with a palpable mass in the left groin area with moderate hardness and no clear boundaries. The local skin had a normal appearance and temperature without erythema, swelling, ulcers, or purulence. The patient’s skin and mucous membranes showed no petechiae or ecchymoses. Factors V and XII levels were decreased, with factor V showing a more marked decrease. His platelet count was also normal. Thus, other hemorrhagic diseases were ruled out. Further, coagulation factors VIII and IX were normal, ruling out both hemophilia A and B. Normal maximum clot strength indicated normal platelet aggregation, which excluded Bernard–Soulier syndrome, Glanzmann thrombocytosis, and other inherited platelet dysfunction diseases. Since both PT and APTT were prolonged and thrombin time (TT) and fibrinogen (Fg) levels were normal (Table 1), we measured the levels of coagulation factors involved in the common pathway, namely factors II, V, VII, and VIII. However, factors II, VII, and VIII, as indicated above, were normal; thus, we focused on the altered factor V levels (Table 2).

**Table 1:** Blood coagulation function test results during hospitalization.

PT (s) (7.4 to 13.4 s)	APTT (s) (16.9 to 36.9 s)	INR	Fg (mg/dL) (200 to 400 s)	TT (s) (14 to 21 s)
67.0	122.7	7.34	300	16.9

Abbreviations: APTT = activated partial thromboplastin time, Fg = fibrinogen, INR = International normalized ratio, PT = prothrombin time, TT = thrombin time.

**Table 2:** Clotting factor levels during hospitalization.

Clotting factors	Value in the patient	Normal range
II	72.1%	70% to 120%
V	5.6%	70% to 120%
VII	69.8%	70% to 120%
VIII	81.4%	70% to 150%
IX	73.9%	70% to 120%
X	57.9%	70% to 120%
XI	63.2%	70% to 120%
XII	34.0%	70% to 150%

**Figure 1.**
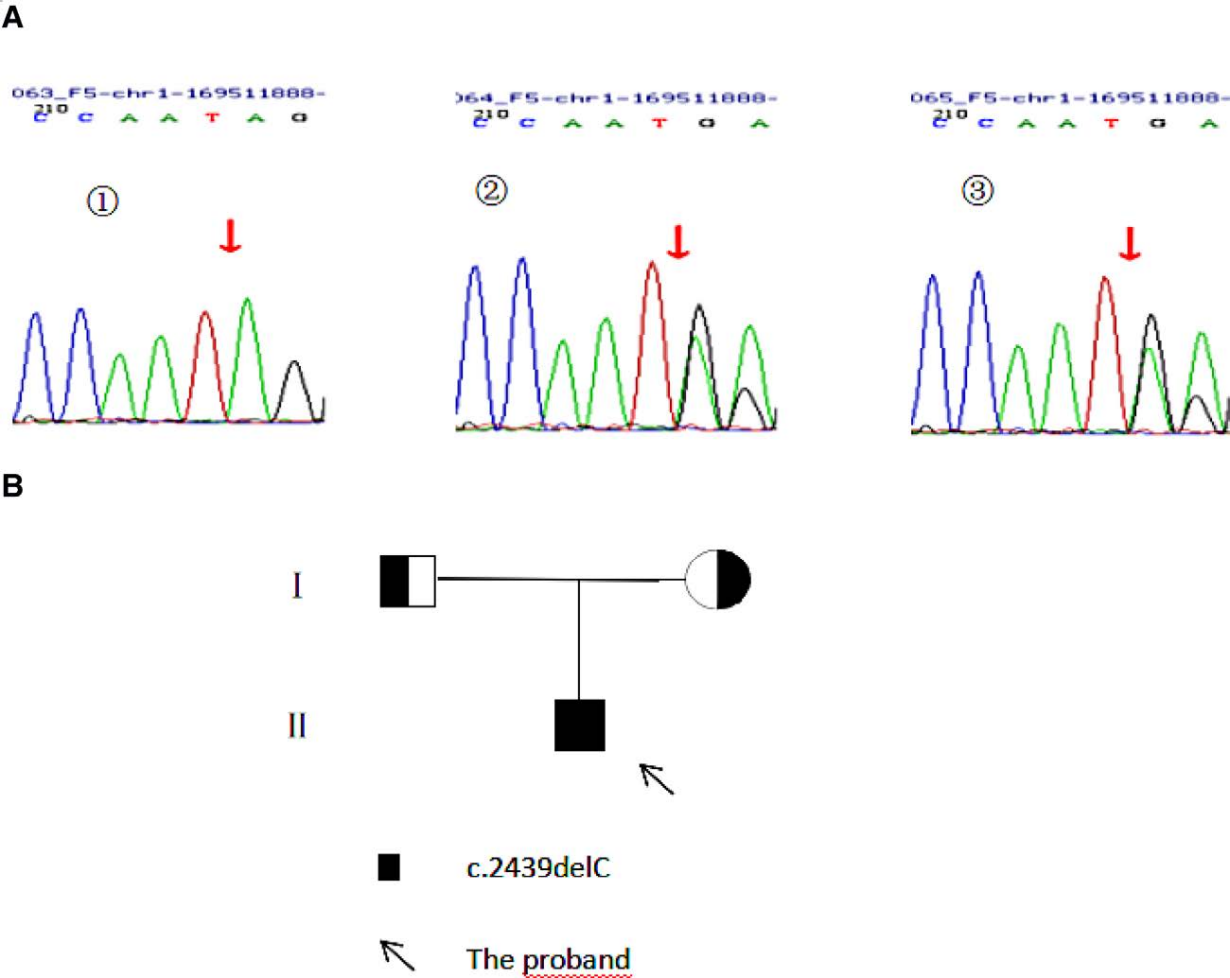
Gene sequencing map of the patient and parents. (A) (1) The arrow indicates the homozygous mutation of the proband’s F5 gene exon 13 c.2439delC (p.I814Lfs*23); (2) the father of the patient, with a heterozygous mutation of c.2439delC (p.I814Lfs*23) in exon 13 of the F5 gene; (3) the mother of the patient, with a heterozygous mutation of c.2439delC (p.I814Lfs*23) in exon 13 of the F5 gene; (B) pedigree of the proband.

Factor V deficiency occurs in liver disease and disseminated intravascular coagulation. Hepatitis B and C viral test results were negative. Fg level, platelet count, hemoglobin level, and liver function were normal (Table 3). Therefore, the patient was considered to have congenital factor V deficiency. The results of the genomic analysis showed a homozygous mutation of the factor V gene (Fig. 1). The homozygous mutation c.2439delC in exon 13 resulted in a frameshift mutation in the amino acid (p.I814Lfs*23). No correlation reports for this locus have been described in the literature. The patient’s parents were first-degree cousins. According to the American College of Medical Genetics and Genomics guidelines, the variant was initially identified as a pathogenic variant, PVS1 + PM2 + PM3_Supporting (hom). Genetic testing confirmed this diagnosis.

**Table 3:** Results of laboratory investigations during hospitalization.

Laboratory test items	Value in the patient	Normal range
PLT	388 × 10^9^/L	100 to 450 × 10^9^/L
HGB	114 g/L	120 to 160g/L
ALT	<8 U/L	<49 U/L
AST	16 U/L	<40 U/L
Addiment C3	1.08 g/L	0.70 to 2.06 g/L
Addiment C4	0.20 g/L	0.11 to 0.61 g/L
Addiment C1q	19.73 mg/dL	15.7 to 23.7 mg/dL

Abbreviations: ALT = alanine aminotransferase, AST = aspartate aminotransferase, HGB = hemoglobin, PLT = platelet count.

The patient received fresh frozen plasma (FFP) infusion (14 mL/kg twice a week). The left iliac fossa mass stopped growing and the coagulation function exhibited a significant improvement (Table 4). Following discharge from our hospital, the patient was recommended to seek medical help before any surgery or following trauma. When a deep hemorrhage is found, timely infusion of FFP should be administered.

**Table 4:** Results of blood coagulation function testing during hospitalization.

PT (s) (7.4 to 13.4 s)	APTT (s) (16.9 to 36.9 s)	INR	Fg (mg/dL) (200 to 400 s)	TT(s) (14 to 21 s)
21.1	42.6	2.13	257	18.0

Abbreviations: APTT = activated partial thromboplastin time, Fg = fibrinogen, INR = International normalized ratio, PT = prothrombin time, TT = thrombin time.

## 3. Discussion

Factor V is a glycoprotein synthesized by hepatocytes and megakaryocytes, also referred to as proaccelerin or labile factor. It functions as a cofactor in the activation of factor X within the prothrombin complex and plays a crucial role in promoting clot formation during the coagulation cascade.^[5]^ Factor V enhances the conversion of prothrombin to thrombin. In addition to its procoagulant activity, factor V exhibits anticoagulant properties through the inactivation of factor VIII mediated by activated protein C.^[6]^

The first patient with a deficiency in factor V was identified in 1943, while the initial mutation responsible for factor V deficiency was discovered over 50 years later.^[7]^ Mutations in the F5 gene encompass missense mutations, premature termination mutations (including small deletions, small insertions, and nonsense mutations), as well as splicing and frameshift mutations.^[8]^ Most nonsense mutations in the F5 gene are predominantly located within the factor V B domain, whereas missense mutations primarily occur in the A and C domains. Additionally, splicing mutations have been associated with more severe defects in factor V function.^[9]^ The only mutation in the factor V A1 domain that leads to type II factor V defects is factor V New Brunswick, which was reported in 1995.^[7]^ Additionally, various single nucleotide polymorphisms exist within the F5 gene. Distinguishing between true mutations and benign polymorphisms can be challenging; therefore, thorough familial investigations are essential. Given the large size of the F5 gene and the absence of mutation hotspots, most mutations associated with factor V defects tend to be unique and confined to a specific family or region.^[10]^ Approximately one-third of genetic defects are attributed to mutations that result in premature termination codons (PTCs). PTCs represent a nonsense-mediated mRNA decay that harbors the translation.^[11]^ It has been well established that mutations resulting in PTCs are correlated with severe manifestations of factor V defects.^[10]^

Congenital factor V deficiency is a rare bleeding disorder with an autosomal recessive inheritance pattern and an estimated incidence of 1 per 1 million in the general population.^[1]^ Patients with factor V deficiency present a wide variety of clinical features, ranging from asymptomatic conditions to life-threatening bleeding. Bleeding events in severe deficiency are usually observed at birth or early childhood. However, there is no established relationship between clinical presentations and residual factor V levels.^[3,12]^ Mucosal bleeding is the most common clinical manifestation. Life-threatening bleeding, including central nervous system and gastrointestinal bleeding, umbilical cord bleeding, and recurrent miscarriages, rarely present in patients with factor V deficiency.^[2,13,14]^ Patients with severe factor V deficiency experience mild to severe bleeding, but many patients with factor V levels < 1% exhibit fewer bleeding episodes than expected.^[15]^ The reason for this phenomenon is unknown; however, residual platelet factor activity may be involved.^[16,17]^

Acquired factor deficiency is caused by factor V inhibitors. Chemical agents, drugs, and surgical procedures are the most common causes of acquired factor V deficiency.^[18–22]^ The severity of the clinical presentation depends on antibody titers and the access of antibodies to platelet factors.

The clinical evaluation of a patient with bleeding symptoms should begin with the taking of a careful medical history and noting the child’s age, sex, clinical presentation, medical history, and family history. Bleeding from the soft tissues, muscles, and joints often suggests hemophilia or other clotting factor disorders. Inherited bleeding disorders should be considered if bleeding manifestations occur congenitally or in early childhood, especially when there is a positive family history.^[23]^ Our patient had a history of recurrent epistaxis in early childhood, but no history of subcutaneous hematoma. Thus, platelet disorders and platelet-vessel interactions were excluded. When the patient was diagnosed with hemarthrosis and coagulation factor disorder, his childhood history of recurrent epistaxis further suggested the possibility of a congenital coagulation factor disorder. Genetic testing further confirmed the homozygous mutation of the factor V gene in this patient. His parents had heterozygous mutations at the corresponding locus. Homozygous mutations are rare, and the patient had no relevant family history. Initially, the patient’s parents had concealed that they were close relatives. Finally, the patient’s parents were repeatedly questioned, and they confessed that they were cousins.

The primary treatment for factor V deficiency is on-demand therapy with FFP to maintain factor V levels above 20%. There are currently no well-defined guidelines for managing patients with factor deficiencies. However, this management depends on different aspects, including residual factor V levels, type and severity of the bleeding event, maintaining a minimum hemostasis level, and type of treatment (Table 5).^[24]^

**Table 5:** Recommended treatment for patients with factor V deficiency.

	On demand	Prophylaxis	Surgeries	
			Major	Minor
Recommended level of FV	10%	10%	>15% to 20%	–
Dosage and kind of treatment	FFP: 15 to 25 mL/kg platelet transfusion	FFP: 20 mL/kg 2 times per week	FFP:15 to 25 mL/kg (before surgery) or 10 mL/kg every 12 h Platelet transfusion (if required)	FFP:15 to 20 mg/kg or 1 g 4 times daily tranexamic acid

## 4. Conclusions

Congenital factor V deficiency is a rare autosomal recessive inherited coagulation disorder. Patients with factor V deficiency exhibit a variety of clinical characteristics. This emphasizes that coagulation factor deficiencies should be suspected in patients presenting with joint masses combined with coagulation dysfunction. Nonetheless, no precise relationship between clinical manifestations and residual factor levels was detected. Genetic testing may help confirm the diagnosis. The factor V gene variant c.2439delC (p.I814Lfs*23) in exon 13 was first reported in this case. The main treatment for factor V deficiency is on-demand administration of FFP.

## Author contributions

**Data curation:** Yifan Zhang.

**Formal analysis:** Chaomin Wan, Yu Zhu.

**Investigation:** Lu Liu, Qin Guo, Yiyuan Li, Shuanghong Luo.

**Methodology:** Chaomin Wan, Yu Zhu.

**Project administration:** Yu Zhu.

**Writing – original draft:** Yifan Zhang.

**Writing – review & editing:** Yifan Zhang, Yu Zhu.
